# In Search of an Imaging Classification of Adenomyosis: A Role for Elastography?

**DOI:** 10.3390/jcm12010287

**Published:** 2022-12-30

**Authors:** Sun-Wei Guo, Giuseppe Benagiano, Marc Bazot

**Affiliations:** 1 Research Institute, Shanghai Obstetrics & Gynecology Hospital, Fudan University, Shanghai 200011, China; 2 Shanghai Key Laboratory of Female Reproductive Endocrine-Related Diseases, Fudan University, Shanghai 200011, China; 3 Faculty of Medicine and Dentistry, Sapienza, University of Rome, 00161 Rome, Italy; 4 Department of Radiology, Tenon University Hospital, Assistance Publique des Hôpitaux de Paris (AP-HP), Sorbonne Université, 75012 Paris, France; 5 Groupe de Recherche Clinique (GRC-6), Centre Expert en Endométriose (C3E), Assistance Publique des Hôpitaux de Paris, Tenon University Hospital, Sorbonne Université, 75000 Paris, France

**Keywords:** adenomyosis, elastography, fibrosis, imaging classification, magnetic resonance imaging, transvaginal ultrasound

## Abstract

Adenomyosis is a complex and poorly understood gynecological disease. It used to be diagnosed exclusively by histology after hysterectomy; today its diagnosis is carried out increasingly by imaging techniques, including transvaginal ultrasound (TVUS) and magnetic resonance imaging (MRI). However, the lack of a consensus on a classification system hampers relating imaging findings with disease severity or with the histopathological features of the disease, making it difficult to properly inform patients and clinicians regarding prognosis and appropriate management, as well as to compare different studies. Capitalizing on our grasp of key features of lesional natural history, here we propose adding elastographic findings into a new imaging classification of adenomyosis, incorporating affected area, pattern, the stiffest value of adenomyotic lesions as well as the neighboring tissues, and other pathologies. We argue that the tissue stiffness as measured by elastography, which has a wider dynamic detection range, quantitates a fundamental biologic property that directs cell function and fate in tissues, and correlates with the extent of lesional fibrosis, a proxy for lesional “age” known to correlate with vascularity and hormonal receptor activity. With this new addition, we believe that the resulting classification system could better inform patients and clinicians regarding prognosis and the most appropriate treatment modality, thus filling a void.

## 1. The Tower of Babel

Featuring the presence of endometrial glands and stroma within the myometrium [1], adenomyosis is a uterine disease that affects many women of reproductive age and contributes to dysmenorrhea, pelvic pain, abnormal uterine bleeding (AUB)/heavy menstrual bleeding (HMB), and subfertility [2,3,4,5]. Consequently, it impacts negatively on the quality of life of the afflicted woman [6]. It is also associated with increased risk of several adverse pregnancy outcomes [3,7]. Due largely to its poorly understood pathogenesis and pathophysiology, its clinical management still poses a challenge [8] and will remain problematic until its various features can be linked to specific clinical symptoms, a difficult task because the symptoms of adenomyosis are not pathognomonic.

Dysmenorrhea, pelvic pain, AUB/HMB, and subfertility/infertility have been reported in 90.8% of affected women [9]; however, these conditions are unfortunately not specific to adenomyosis, and, as such, the diagnosis has to rely on other means [10]. The diagnosis of adenomyosis used to be determined solely by histology after hysterectomy, and, as such, it was found disproportionately in older, parous or perimenopausal women, with a prevalence ranging wildly from 10% to 88% [11]. With the advent of modern imaging technology, adenomyosis is increasingly diagnosed in a non-invasive fashion [12,13]. In the last three decades, transvaginal ultrasonography (TVUS) and magnetic resonance imaging (MRI) have gradually taken over the role of the post-hysterectomy histological evaluation as the principal diagnostic tool, and become the major diagnostic tools for adenomyosis.

For this reason and given the wild variation of adenomyosis in terms of location, pattern, size, and affected area, the one pressing issue is to classify the disease based on imaging diagnosis. Ideally, an established classification system should link the extent of the disease with symptoms, their severity, and/or prognostic indices [14,15,16]. It also should help to understand the pathogenesis, pathology, clinical manifestations of adenomyosis, suggest the best treatment modality, provide a prognosis, and if at all possible be capable of monitoring treatment response. Finally, since adenomyosis is still an under-investigated disease, a widely accepted classification system should allow improved comparisons of research data and clinical outcomes [17,18].

Due to these pressing needs, several image-based classification systems have been proposed [5,14,15,19,20,21,22] and summarized [23], but so far, no consensus has been reached. Recognizing the importance of an agreed classification, available information has been condensed in a number of reviews published over the last 15 years [5,15,16,23,24] and renewed efforts have been made towards a non-invasive ultrasound classification [25]. Unfortunately, so far, consensus has not been reached and without a consensus classification system, there is a real danger of repeating the catastrophic fiasco of building the Tower of Babel.

Hopefully, the increasing interest in developing an agreed classification will soon overcome the existing objective difficulties. Here we propose a possible way to escape from this impasse.

## 2. Early Attempts to Classify Adenomyosis

Early attempts to diagnose adenomyosis used histological evaluation, with the intention to quantify the depth of endometrial invasion within the myometrium both to gauge the extent of the disease and to distinguish a cut-off point for diagnosis. A major issue has been the critical disagreement on the definition of adenomyosis using histology as a gold standard reference point [24]. Yet, a uniformly consensus reporting system may be a first step for a classification that takes symptoms into account [23].

One important source of disagreement is represented by the depth to which the mucosa needs to ‘invade’ the myometrium before a diagnosis of adenomyosis is made. The issue is complicated by at least two variants. The first is the presence of irregularities at the endometrial-myometrial interface (EMI), with the ensuing need to agree on a cut-off point to dichotomize normality and pathology. The second challenge is the often patchy distribution of adenomyosis within the myometrium, with a critical disagreement on how to define adenomyosis using histology as a gold standard reference point [24]. These uncertainties are reflected in the frequency of diagnoses reported by histopathologists with variations which can be as high as 9-fold [26].

An important driver has been the need to minimize or remove the risk of over-diagnosis. The distinction between adenomyosis and the normal variation at the EMI still poses a challenge. This is particularly the case because of the difficulty researchers encounter when attempting to establish the link between histological features and symptoms. It is perhaps not surprising that researchers have proposed different cut-off points. On the other hand, because hysterectomy is typically performed on symptomatic women, a stringent cut-off threshold risks under-diagnosis.

The introduction of imaging-based diagnosis has permitted a global view of the uterus, providing an opportunity for disease mapping that is much less invasive and less labor-intensive as compared to histological diagnosis. A distinction, however, needs to be made between disease mapping based on anatomical location of lesions and disease categorization based on symptom and its severity. Each of these has its own challenges. Published literature mostly reports on the accuracy of diagnosis based on whether adenomyosis is present or absent, rather than the accuracy of each individual feature, location, or cut-off points for diagnosis.

Another feature that has attracted debate in relation to the classification of adenomyosis is the relation between the ‘classic’ uterine adenomyosis and ‘endometriotic’ lesions in the pouch of Douglas and uterovesical pouch that exhibit histological similarities to adenomyosis. One unresolved issue is about their pathogenesis and whether they originate from spread of uterine disease to surrounding tissues or from spread of endometriosis into the serosa, and this could have important implications when it comes to classification.

Understanding the pathophysiology can have implications for classification. Adenomyosis could represent a spectrum of diseases with the commonality of the presence of ectopic endometrial epithelium and stroma within the myometrium [27,28]. In principle, these debates can be settled by establishing a phylogenetic relationship between adenomyotic lesions and their neighboring endometriotic lesions based on genomic or epigenomic sequence data [29].

When attempting to create a classification of adenomyosis, an important consideration is the place of junctional zone (JZ) hyperplasia and whether it is pathognomonic of adenomyosis when it exceeds 11 mm. Although there is no consensus on the existence and importance of JZ hyperplasia, it was defined as partial or diffuse thickening of the JZ from 8 to 11 mm in the absence of additional imaging signs of adenomyosis [23]. Initially it had been held that the presence of a JZ thickness of more than 11 mm would be pathognomonic of adenomyosis [30] and its measurement has been commonly considered as an indirect way to diagnose the disease. However, today caution is recommended when the JZ is used alone as a diagnostic procedure [17,31,32].

Rasmussen et al. tried to classify adenomyosis when confined to the inner myometrial and JZ regions into three separate TVUS-based categories: ‘adenomyosis of the inner myometrium’; ‘junctional zone disease’, characterized by a serrated appearance of the JZ; and ‘linear junctional zone’ [33]. This interesting study raises concerns due to the high variability in assessing JZ even when using 3D TVUS examination. Other researchers have suggested that adenomyosis could be divided into different categories based on morphology as well as location of the lesion. In this connection, the subdivision into two variants has been proposed. The first, termed ‘diffuse adenomyosis’, is applied to lesions that affect more than one myometrial wall and it could be symmetric or asymmetric. A further subdivision into three sub-categories according to the depth of involvement reaching less than one-third, less than two-thirds, or greater than two-thirds of the myometrium has also been suggested [22]. The second variant is ‘focal adenomyosis’, defined as a single circumscribed mass within the myometrium [34]. Finally, ‘adenomyoma’ features a solid or cystic myometrial mass with indistinct margins of primarily low-signal intensity on T2-weighted MRI sequences; it is normally located in the mid-myometrium and rarely protrudes into the endometrial cavity or under the serosa [22]. A new subtype, ‘external adenomyosis’ (anterior or posterior), has been introduced for the above-mentioned lesions found adjacent to the uterine serosa, being significantly associated with pelvic or deep endometriosis [35].

## 3. The Current State of Diagnostic Tools

To overcome the heterogeneity and to help improve the diagnostic accuracy of TVUS, an international expert panel published in 2015 the Morphological Uterus Sonographic Assessment (MUSA) consensus statement on the descriptive markers for the diagnosis of adenomyosis on TVUS, with the goal of providing a standardized terminology for describing ultrasound images of normal and pathological myometrium [36]. The same group also published a consensus on the standardized classification and reporting of adenomyosis based on TVUS [37], and, recently, a consensus statement on revised definitions of MUSA features of adenomyosis was published [21]. These now consist of two groups: direct and indirect features. Direct features include the presence of myometrial cysts, of hyperechogenic islands, or of echogenic subendometrial lines and buds, each of which is indicative of the presence of adenomyotic lesions in the myometrium [21]. In contrast, indirect features are secondary to the presence of lesions in the myometrium, such as muscular hypertrophy (globular uterus) or artifacts (e.g., shadowing), which are merely suggestive of adenomyosis in the absence of direct features [21]. Despite these updated and revised features, the importance of each individual ultrasound feature of adenomyosis remains unclear [21].

Imaging has demonstrated acceptable accuracy, although the diagnostic precision for individual features is less evident [5] and the necessary skill and expertise are not universally available despite advances. There are additional complexities. Currently, TVUS is the first-line technique in gynecological work-up due to its wide availability, non-invasiveness, real-time capability, and being easier than MRI to operate as well as less expensive than MRI, and it also permits a dynamic examination to gauge organ mobility and site-specific tenderness. Through two-dimensional (2D) and 3D setting and the color flow Doppler version of TVUS, a good view of the uterus and its pathology can be obtained, except in the presence of numerous associated large uterine leiomyomas [38,39]. Compared with TVUS, transabdominal ultrasonography has limited value, but can be an effective alternative when the vaginal route is inaccessible, or in the case of a grossly enlarged uterus [38]. Compared with 2D and 3D TVUS, color flow Doppler ultrasonography has the added advantage of providing information on the location, amount, and type of blood flow [40,41]. This can help to differentiate adenomyosis from uterine fibroids, thereby enhancing the overall diagnostic accuracy [39]. In addition, Doppler ultrasound can discriminate vessels from myometrial cysts and ectopic foci of adenomyosis and vice versa. In the hands of a trained sonographer, TVUS is also quite accurate in diagnosing gynecological pathologies, including adenomyosis.

MRI is also useful in identifying the location, number, and the extent of adenomyotic lesions. In contrast to 3D TVUS, the zonal anatomy of the uterus is clearly demonstrated on T2-weighted MR images, although zonal anatomy as seen through MRI and TVUS differs [42]. Even to untrained eyes, it provides clear pictures of the pelvic anatomy and the uterus, in either sagittal, or coronal, or transverse plane, and in slice by slice. Because of its limited availability and higher cost, however, it is often employed as a second-line work-up, especially after inconclusive TVUS investigation [43]. Compared with TVUS, MRI provides more detailed intrapelvic information, allowing concurrent diagnosis of ovarian endometrioma and deep endometriosis [44]. In addition, it has superior objectivity when diagnosing adenomyosis [45].

The pooled sensitivity and pooled specificity of MRI for diagnosing adenomyosis is 77% and 89%, respectively, slightly better than that of TVUS (72% and 81%). However, it is less operator dependent and more objective, and it relies less on the capacity of the observer to diagnose [46].

Thus, both TVUS and MRI are acceptable imaging modalities for diagnosing adenomyosis. In terms of accuracy, however, there is still ample room for improvement. For one thing, the substantially heterogeneous diagnostic criteria used in TVUS certainly do not help to enhance the edge of TVUS [14,31]. This is due, perhaps in no small part, to the fact that many features used in either TVUS or MRI in diagnosing adenomyosis are essentially morphometric measurements, which may depict lesion as well as tissue geometry and/or topography that are influenced conceivably by lesional microstructure and pathology, which simply are out of reach by either TVUS or MRI.

## 4. Imaging-Based Classification of Adenomyosis

Some classification systems have already demonstrated their utility in helping to understand adenomyosis better. For example, the Kishi system has helped us to understand that intrinsic adenomyosis, lesions that are confined to the subendometrial layer without involving the outermost myometrium, is often associated with the history of iatrogenic uterine procedure [19]. In addition, intrinsic adenomyosis is often associated with HMB while extrinsic or external adenomyosis is associated with pain [47].

Recently, Exacoustos et al. investigated the relation between an ultrasound-based disease classification and symptoms [25]. Women with ultrasound diagnosis of diffuse adenomyosis were older and had heavier menstrual bleeding compared to those with focal disease, but, when it came to the severity of dyspareunia and dysmenorrhea, there was no statistically significant difference. In contrast, focal adenomyosis was associated with a higher percentage of infertility. Overall, no direct correlation between ultrasound depictions of the extent of the disease and symptoms was found. The authors speculate that this may be related either to co-existent pathology, or to a true lack of correlation between symptoms and disease extent. However, better statistically powered studies will be warranted before definitive conclusions can be reached [25].

Table 1 summarizes various proposals for imaging-based classifications of adenomyosis. One conspicuous feature shared by all these proposals is that all of them attempted to correlate various classification parameters with either possible pathogenesis, or symptomatology or its severity. However, none of these parameters has been shown to be linked with the histological features of endometriotic lesions [23].

All imaging classification systems proposed so far [5,14,15,19,20] are sensible. For example, part of Bazot and Darai’s system [14] as well as that of Kishi’s [19] can be traced back to the original Sampson’s grouping according to the origin or pathogenesis: invasion from within the uterus (arising from the uterine inner myometrial layer); invasion from outside the uterus (arising from the uterine outer myometrial layer); and misplaced endometrial tissue in the uterine wall (possibly arising from embryologically, solitary pluripotent Müllerian remnants) [14,20,48]. The intrinsic/internal type of adenomyosis also makes sense, since as early as 1908 Cullen was able to establish the physical continuity between eutopic and ectopic endometrium in many patients with adenomyosis [16,49]. This is demonstrated, using modern technology, by 3D rendition showing that adenomyosis lesions are stereoscopically characterized by an “ant colony-like network” that connects directly with endometrial glands [50].

These proposed classification types are a welcome step towards a homogenized system so that all gynecologists may speak the same language while some of them have been shown to be useful in separating different phenotypes. However, none of them has been shown to be able to stage adenomyosis, to correlate the severity of symptomology or have prognostic value. At best, a filing system can be established, upon which a patient could be classified into one of the classification categories, as shown recently [51].

Therefore, we need to ask ourselves: Can we push the envelope of current imaging modalities?

## 5. The Physical Limits of TVUS and MRI

The MUSA consensus on the standardized classification and reporting of adenomyosis based on TVUS proposes inclusion of lesional location, distinction between focal and diffuse adenomyosis, identification of cystic/non-cystic elements, and involvement of the myometrial layer grouped into three types: inner/sub-endometrial myometrium (Type I), middle myometrium (Type II), and outer/sub-serosal myometrium (Type III). In addition, the disease extension is classified as mild, moderate, or severe, and measurement of lesion size [37] (Table 1).

While these efforts will undoubtedly help improve the diagnostic accuracy of TVUS, few have ever questioned whether the current TVUS instrumentation/technology, and perhaps MRI as well, may have reached its physical limit, especially because many features of the imaging findings, including the revised ones [21], bear little correlation with either symptom severity, or underlying lesional pathology, or prognosis. After all, there will always be limits to human discoveries, always with issues that are ultimately unknowable, undoable, or unreachable [52]. This actually occurred in diagnosing deep endometriosis when lesions were small [53].

Raising this question and confronting it squarely can be sobering and helpful, since this would prompt us to think of possible solutions and other options, such as sonohysterography, hysteroscopy, elastography, or their combination [5,54].

More fundamentally, TVUS detects adenomyosis based on the hypo- or hyper-echogenic appearance and/or the shape, symmetry/asymmetry, the integrity of the JZ, vascularity, as well as acoustic “shadowing” effect caused by lesions [21]. MRI, on the other hand, detects adenomyosis through distinguishing tissue structures based on their water content via multiplanar imaging and excellent soft tissue contrast. Presumably, these features are determined by lesional microscale characteristics, such as the composition of epithelial/stromal cells, extracellular matrix (ECM) stiffness, fibrotic content, etc., which cannot be quantitated by either imaging mode. In particular, tissue elasticity is a fundamental biologic property that directs cell function and fate [55,56,57], but unfortunately the tissue properties that are detected by either TVUS or MRI tell us nothing about the distinct hardness of pathological tissues, which can be detected by palpation, an approach used since ancient times.

In addition, once an affirmative diagnosis of adenomyosis, by either TVUS or MRI, is reached, an immediate question would be: What is the best treatment modality for the patient? Surgery? Thermal ablation? Or medication? These questions become all the more complicated if the patient’s wishes, as well as the indications and contraindications of a specific modality are also considered.

Apparently, within the TVUS or the MRI realm, these questions are difficult to answer. Therefore, we need to open-mindedly seek other options.

## 6. The Case for Incorporation of Elastography to Move Forward

To address these issues, the imaging findings should closely reflect the lesional development stage, as well as the extensiveness of adenomyosis and co-existing pathology. Since fibrogenesis underpins the lesional progression, the extent of fibrosis can be viewed as the ultimate destiny of adenomyotic lesions. In other words, fibrosis is one important pathognomonic feature of adenomyosis, as in endometriosis [58,59]. Incidentally, uterine fibroids also have excessive ECM deposition [60], and, perhaps to a lesser extent, endometrial polyps [61], intrauterine adhesions [62], and even the ovaries [27,63] and endometrium [64] could undergo a process of increasing fibrosis. The extent of fibrosis in either adenomyotic lesions or fibroids conceivably determines the lesional stiffness or rigidity, which could be evaluated by palpation through tissue deformation. Hence, lesional stiffness contains information inherently embedded within adenomyotic lesions, revealing just how advanced the lesion is. Unfortunately, neither TVUS nor MRI can be used to evaluate this important attribute.

Elastography is an emergent imaging technology and only recently the ultrasound-based version became commercially available, although only with high-end ultrasound instruments. It generates images of tissue rigidity or stiffness, by ultrasound elastography (UE) [65] or magnetic resonance elastography (MRE) [66]. Thus, it is akin to the traditional palpation in clinical examination but is less subjective, requires little experience, and provides better spatial localization information [67]. Currently, the application of MRE in gynecology has been scanty, but UE has become more and more popular [68,69,70]. The greatest strength of elastography is the fact that it can characterize tissue biomechanical properties (i.e., viscoelasticity) non-invasively, something that neither computer tomography (CT), ultrasound or MRI can [67]. In addition, it has a much wider dynamic range than CT, ultrasound, and MRI [66].

To appreciate the importance of the width of the dynamic range of a detector, perhaps the invention of the spectroscope would be a good example. Sir Isaac Newton invented the triangular prism, which for the first time decomposed sunlight into a spectrum of light with different colors or wave lengths, effectively increasing the dynamic range of our visualization of light. Based on this increased dynamic range endowed by the prism, German chemist Robert Benson and physicist Gustav Kirchhoff were able to invent jointly the spectroscope, which can determine the chemical components of an unknown sample by the light emitted when a sample is burned. With the expanding range of wave lengths to include infrared, ultraviolet, and even X-rays, the spectroscopes can further become spectrometers, greatly enhancing the ability, scope, and dimension of detection.

UE can be grouped roughly into two different categories: strain imaging and shear wave imaging [67]. Both methods require mechanical excitation, which is akin to applying a force in palpation. Depending on the excitation methods, measured physical quantity, and the method of displaying the measured quantity, UE can be further divided into different groups [67], such as transient elastography (TE) and acoustic radiation force impulse (ARFI) [65]. Strain UE measures the tissue deformation or displacement generated by applying pressure (as an excitation) with a probe on the body surface, while shear wave UE records the propagation of shear-waves after excitation. In many commercial UEs, the tissue stiffness is displayed in a false-color image overlaid on B-mode images, often side by side with the B-mode image, which greatly facilitates the interpretation of imaging results.

**Figure 1:**
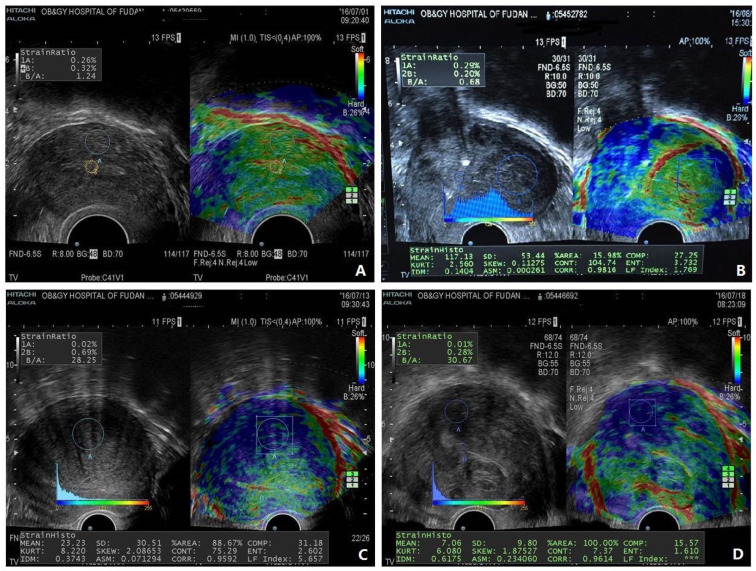
Ultrasonic strain elastographic pictures showing a normal uterus (**A**), uterine fibroids (**B**), diffuse adenomyosis (**C**), and focal adenomyosis (**D**). In (**A**), the normal myometrium shows the greenest and the less yellow color represents mild stiffness of the tissue. In (**B**), the uterine fibroid shows the greenest and the less blue color represents medium stiffness of the tissue, and there is also a typical red color pseudocapsule circling around the fibroid. In (**C**), diffuse adenomyotic tissue shows the broad area of blue color representing the even stiffer tissue. In (**D**), focal adenomyosis with co-occurrence of uterine fibroid can be seen. Focal adenomyotic tissue (the upper left area) shows the local area of blue color representing the even stiffer tissue. The uterine fibroids (the middle lower area) show a typical red-colored pseudocapsule circling around the fibroid. Typical serosal surface of uterus shown in red color could be seen in every uterus. The color bar shown on the upper right corner in each figure is the color key, showing that the tissues with the red color are the softest while those with the blue, the hardest or most rigid. The cycles indicate the region of interest (ROI), and various parameters are shown at the bottom of the figure, which are combined with the LF index, where the stiffness index, originally designed for gauge liver function (thus, “LF”), in this case represents the relative stiffness. It can be seen that both the B-mode image and the elastographic image are displayed side by side. Abbreviations used: AM: adenomyosis; Normal: normal uterus. Replicated from Liu et al. [71] (Reprinted with permission from Reproductive Sciences).

**Figure 2:**
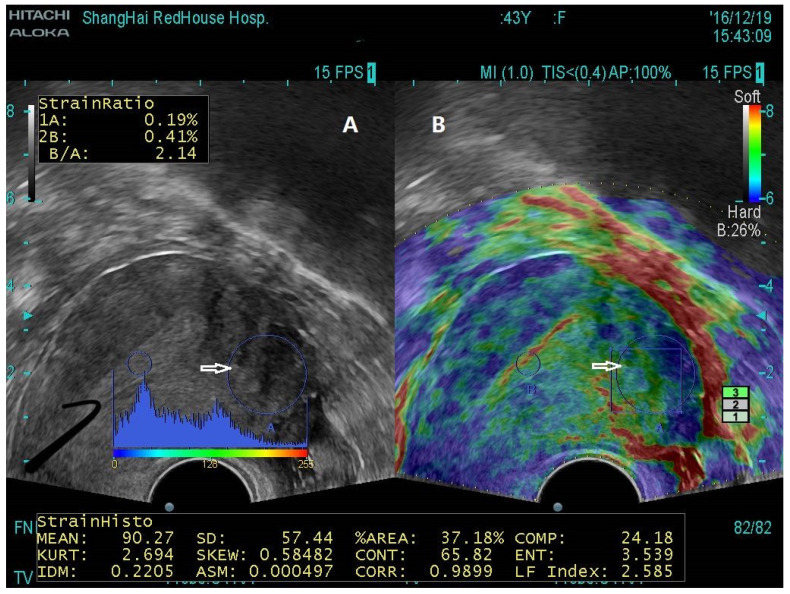
Transvaginal ultrasound image for a patient with a small uterus (59 × 58 × 57 mm) who complained of moderate dysmenorrhea with elevated CA125 level and was suspected with AM. (**A**) The big, circled area (white arrows) showed the ROI. The conventional B-mode TVUS image showed no sign that was consistent with a typical or spherical enlarged uterus or the presence of mild but not severe or obvious internal inhomogeneous echo in ROI. (**B**) Transvaginal elastosonography image showing an increased stiffness value (LFI = 2.585) in the same ROI shown in the TVUS (white arrow), indicative of adenomyosis. AM indicates adenomyosis; LFI, liver function index; ROI, region of interest; TVUS, transvaginal ultrasound. Replicated from Liu et al. [71] (Reprinted with permission from Reproductive Sciences).

**Figure 3:**
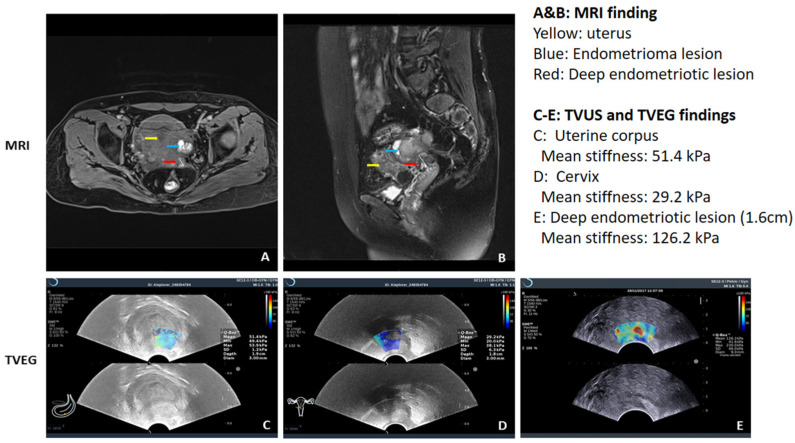
The use of ultrasonic elastography to diagnose deep endometriosis and its consistency with MRI findings. For this patient who complained of severe dysmenorrhea, both MRI (**A,B**) and a shear-wave ultrasonic elastography (**C**–**E**) were used to diagnose deep endometriosis. While the anatomical localization of the deep endometriotic lesion is not as straightforward as MRI, the elastography gave a lesional stiffness value, and in this case the lesion is quite stiff and thus highly fibrotic. Note that for this shear-wave elastography which gives out absolute tissue stiffness value in kilo Pascal (kPa), the color key is reversed, with the blue color depicting the softest while the red color indicating the hardest tissues. More detailed explanation for each figure is given at the upper right panel. (Courtesy of Dr. Ding Ding).

UE has been shown to be used to diagnose adenomyosis [71,72,73,74,75], uterine fibroids [76,77], deep endometriosis [53], and various endometrial pathologies [78]. Remarkably, in adenomyosis, the average lesional stiffness seems to be significantly higher than in uterine fibroids, which, in turn, is higher than in normal myometrium [71,79,80] (Figure 1). More importantly, lesional stiffness, as measured by UE, correlated positively with the extent of lesional fibrosis but negatively with lesional staining levels of PR and vascularity [53,71] (Figure 2). Finally, UE has been shown to enable a proper diagnosis of deep endometriosis (Figure 3).

Figure 1 demonstrates the typical UE images of normal uterus, uterine fibroids, and diffuse and focal adenomyosis. Figure 2 shows both UE and B-mode TVUS figures in a side-by-side manner. Figure 3 shows the use of shear-wave UE in diagnosing deep endometriosis and its consistency with the MRI finding. When lesional stiffness is quantitated by UE, the possible lesional response to hormonal treatment can be properly determined, since lower PR expression is associated with poor response to progesterone treatment [81]. Women with “soft” lesions may be more likely to respond to dienogest treatment simply due to more cellularity and vascularity (thus easier for drug delivery to target tissues/cells), less epigenetic aberrations, and likely higher hormonal response [82]. Therefore, UE could not only improve diagnostic accuracy but, more importantly, help gynecologists to decide the best treatment modality.

Aside from the enhancement in diagnostic accuracy and its use in helping to choose the best treatment modality, the extent of lesional fibrosis/stiffness in both ovarian endometriomas and deep endometriosis correlated positively with the severity of dysmenorrhea [53,63]. Furthermore, the extent of tissue fibrosis in the ovarian cortex adjacent to ovarian endometrioma has also been reported to be correlated negatively with the serum anti-Müllerian hormone (AMH) levels [63].

Moreover, in women with adenomyosis, lesional stiffness has been found to be correlated with severity of dysmenorrhea and the amount of menstrual blood loss (MBL), and can be used to distinguish adenomyotic lesions from uterine fibroids [71]. In fact, the lesional stiffness correlated positively with the amount of MBL [83]. UE can also be used to diagnose deep endometriosis [53], which often co-exists with adenomyosis. More importantly, the lesional stiffness as measured by UE correlated with the extent of tissue fibrosis not only in lesions but also in adjacent EMI and eutopic endometrium [83].

Since lesional fibrosis propagates into neighboring EMI and endometrium [83], and endometrial fibrosis inhibits prostaglandin E2 (PGE2) and hypoxia signaling that is necessary for endometrial repair [83,84], the tissue stiffness as well as extensiveness in the EMI and endometrium could be potentially measured by shear-wave UE, raising the possibility of providing a more objective measurement of the extent of impaired endometrial repair and hence MBL. In light of the close relationship between adenomyotic foci/endometrial stiffness/fibrosis and the amount of MBL, a more objective assessment, by elastography, of the endometrial repair potential may be within reach in the future. Monitoring treatment responses to therapeutic interventions may also be possible.

Since UE have a built-in B-mode ultrasound capability, switching back and forth between UE and traditional ultrasound can be as easy as one mouse click, although TVUS is performed using 3D, in contrast to UE which uses 2D, allowing better evaluation. Therefore, TVUE can be used to diagnose adenomyosis and concurrent gynecological pathologies, such as uterine fibroids, ovarian endometrioma, deep endometriosis, and uterine polyps [53,76,78,85].

More importantly, since other reproductive organs, such as endometrium and ovary, can also show pathological or age-dependent fibrosis and thus increasing tissue stiffness [64,83,86], they can also be evaluated by UE [87,88].

If we distill and combine all the essentials from the MRI-based systems proposed by Kishi et al. [19], Gordts et al. [5], Bazot and Darai [14], and Kobayashi and Matsubara [20], it can be seen that the current systems contain six domains: (1) affected area (internal, external, etc.); (2) location (anterior, posterior, fundal, etc.); (3) pattern (focal or diffused); (4) size (myometrial involvement, <1/3, <2/3, or >2/3); (5) type (cystic or muscular); (6) comorbidity with other gynecological conditions. Here, diffuse adenomyosis is defined as endometrial tissues found diffusely within the myometrium. In contrast, focal adenomyosis is characterized by a single adenomyotic lesion in a given area within the myometrium.

We would argue that the type or cysticity may not be important enough to justify its inclusion in the classification since all adenomyotic lesions (with exception of stromal adenomyosis in postmenopausal women) contain glands (dilated, or not, hemorrhagic, or not, visible, or not, on imaging). Therefore, the appellative ‘cystic’ should be restricted to the rare condition termed “cystic adenomyoma” [89,90]. Cystic adenomyoma should now be distinguished from a new uterine disease entitled accessory cavitated uterine mass [91].

Among the five remaining domains, only size can be quantitated by ordinal and numeric values, assuming cystic adenomyosis is “younger” than the muscular variants. The other four domains are actually nominal or categorical labels, without much information regarding whether a given label (say, left lateral) is clinically more important or meaningful than the other (say, right lateral, or fundal). In other words, within each of the four domains, the system only provides a label without knowing whether one label signals a more or less serious outcome of clinical significance than the other. Simply put, it is just a labelling, and whether or not it has any clinical significance requires further investigation. Since the trend today is to present a summary index, such as the Breast Imaging Reporting and Data System^®^ (BI-RADS^®^) [92], various FIGO staging [86], or the Endometriosis Fertility Index (EFI) [93], which is a uni-dimensional number that correlates with either the disease severity or prognosis (e.g., the 5-year survival rate, or the probability to achieve pregnancy), the current classification systems, although useful, still have a lot of room for improvement. Of particular importance, classification systems such as BI-RADS not only include a lexicon of descriptors and a framework for data collection and auditing but also a recommended reporting structure with a final assessment and accompanying management recommendations, which are very informative and helpful to clinicians.

Importantly, while some labeling may be useful in relation to clinical presentation and/or for pathogenetic purposes (say, the affected area), none of the systems contain any known information regarding the developmental stage (i.e., “age”) of the disease, or possible response to treatment. An ideal image-based classification system should integrate some known molecular, cellular, or histologic markers to correlate with severity of symptoms, and to provide important insights into prognosis and treatment response.

In this regard, incorporation of elastographic evaluation of adenomyotic lesions and possibly their neighboring EMI and endometrium will provide a clinically meaningful, relevant, and informative addition, since it gives the stiffness or the extent of fibrosis of lesions, the EMI, and the neighboring endometrium. In particular, measuring the stiffest of all lesions may enable the determination of how well the patient is going to respond to hormonal treatment.

Thus, in a new classification system, we should include the following: (1) affected area (internal, external, intramural, or full-thickness involvement); (2) pattern (diffuse or focal); (3) the stiffest value of adenomyotic lesions; (4) measurement of the stiffness of the endometrium adjacent to lesions; (5) other pathologies, including the stiffness measurement for deep endometriosis, leiomyoma, or intrauterine adhesion—if any. Of lower importance is the location information, since how well the lesions are going to respond to hormonal treatment should be decided by the hardest lesion irrespective of the location. Finally, when adding information about the areas affected (especially internal vs. external) and the pattern (focal vs. diffuse), we could replace the type of lesion (i.e., muscular, or cystic) with lesional stiffness as measured by UE. The stiffness of the endometrium could inform us of the potential for endometrial repair (the hypoxia and PGE2 signaling).

Obviously, before UE could be included in a new classification there should be extensive validation in patients with adenomyosis of variable clinical presentations and across all racial and ethnic populations, so that we can see how this system can inform clinical decision-making and predict the impact of interventions.

That should then constitute a most informative classification system that tells not only the “age” of adenomyotic lesions, but also their impact on endometrium and ovary, and associated pathology.

## 7. Emerging Problems with the Utilization of Elastography

Despite the great potential for the inclusion of elastography in a new classification of adenomyosis, there may be some doubts about and barriers to the adoption of this incorporation. First, since strain is a relative index of stiffness and changes in proportion to the amount of compression, the tissue stiffness measurement reading may depend on the amount of pressure that the operator exerts on the transducer. In other words, the results based on strain elastography may be thought to be operator dependent. In addition, despite strain imaging having advantages of a short learning curve and wide application across almost the whole body, one conspicuous limitation is that it is a non-quantitative technique and does not provide an absolute stiffness value but rather measures elasticity relative to adjacent regions [65]. Moreover, because of attenuation of vibration energy during propagation, a drop in accuracy can occur when examining deeper tissue regions as compared with superficial ones [65]. Proper training as well as the help of the built-in pressure indicator on the machine should help to minimize inconsistencies. In contrast to strain elastography, shear wave elastography (SWE) can provide an absolute stiffness value and is much less operator dependent.

Second, the stiffness measures used in some commercial ultrasound strain elastography machines do not provide an absolute reading for tissue stiffness. Instead, they only give a so-called liver function index or LFI, which is a built-in stiffness measurement implemented in some machines and is displayed automatically after the ROI is positioned. Based on a regression equation of 11 co-variables derived from machine readings, such as mean, standard deviation, kurtosis, degree of strain, and several quantitative characteristics that indicate the contrast, homogeneity, complexity, and uniformity and direction of tissue texture, LFI was designed to predict the extent of liver fibrosis. The regression equation, initially derived by Tatsumi et al. [94,95] and later refined by Fujimoto et al. [96], was tasked to gauge the extent of *liver fibrosis* and to correlate with the *liver function* in particular. Even though LFI correlated positively with the extent of lesional fibrosis in adenomyosis [71], it was designed to measure liver fibrosis and function, not the extent of fibrosis in adenomyosis or endometrium per se. Consequently, it may not be perfectly optimal for measuring uterine stiffness. In principle, this issue can be addressed by extensive studies in the future to come up with a uterus-specific index measure.

Finally, as ultrasound dissipates very rapidly within a human body, UE may not provide a very accurate reading of the tissue stiffness when the ROI or the lesion is deep underneath the body surface, especially when the ROI or lesion is small in size. Of course, this is not the problem that only UE faces. Other TVUS or transrectal ultrasound have the same limitation.

As a new emerging imaging technology, elastography is still evolving and evolving rapidly [65,97]. Even as of now, UE has been proven to be valuable in enhancing the diagnostic accuracy and in expanding the utility of ultrasound in clinical settings [65]. It has been demonstrated to be able to help diagnose endometrial cancer [98], predict lymph node metastasis [99], and monitor treatment response [100], among other things. In addition, magnetic resonance elastography (MRE) is developing rapidly [97], and MRE has been shown to be able to link MRE findings with functional outcomes [101].

## 8. Conclusions

Through imaging tissue biomechanics, elastography adds a completely new dimension to the detection of adenomyosis and its associated pathology. This is primarily due to the fact that the resultant imaging reflects the *composition* and *organization* of the microstructure underlying uterine pathology in general and adenomyosis in particular. Since the elastographic imaging is overlaid on traditional B-mode images, it also provides readouts on the geometry and topography of adenomyotic lesions just as traditional TVUS. Its importance and significance to imaging diagnosis can be analogous to what palpation is to gynecologic examination. It has a wider dynamic detection range than either CT, ultrasound, or MRI, and, as such, permits small but clinically significant differences between healthy tissues and pathological ones to be discerned. Therefore, it should also improve diagnostic accuracy.

It reads out the tissue stiffness, which is a surrogacy for the “age” of adenomyotic or endometriotic lesions. Since mechanical forces and properties influence cellular behavior and function, the stiffness, as measured by elastography, is intrinsically related to the lesional phenotype. Hence, quantification of the lesional stiffness, and thus the extent of lesional fibrosis, has important implications for staging adenomyosis, predicting prognosis, helping to choose the best treatment modality, and assessing treatment response. These attributes are currently lacking for TVUS and MRI.

In summary, diagnostic imaging by elastography provides not only the same amount of information as TVUS and MRI but also extra information that can be used to choose the best treatment modality for the patient. Its ability to diagnose adenomyosis, uterine fibroids, endometriosis, and endometrial polyps all at once is also an advantage.

Granted, the current elastography technology still has room for improvement, but MRE is on the horizon and UE machines will surely be improved. However, with what we have now, the inclusion of elastography in a classification system for adenomyosis will undoubtedly help better classify and improve patient care. We should eagerly and cautiously embrace this technology with open arms.

## Figures and Tables

**Table 1:** Imaging-based classifications of adenomyosis proposed so far.

Author(s) and Year of Publication	Imaging Platform	Proposed Classification	Rationale	Remarks
Kishi et al. (2012) [19]	MRI	Four subtypes: I: intrinsic; II: extrinsic; III: intramural; IV: indeterminate	Based on Sampson’s observation as well as clinical observations	Subtypes I and II appear to have different pathogenesis, symptomology, and severity
Van den Bosch et al. (2015) [36] Revised in Harmsen et al. (2022) [21] (The MUSA standard)	TVUS	Direct features: Cysts, hyperechogenic islands, echogenic subendometrial lines and buds. Indirect features: Asymmetrical thickening, globular uterus, irregular JZ, fan-shaped shadowing, translesional vascularity, interrupted JZ.	Based on expert consensus through several rounds of modified Delphi procedure	A welcome step towards the establishment of standardized terminology, with the goal to build a uniformly accepted or validated system to diagnose or classify the severity of adenomyosis based on imaging findings.
Bazot and Darai (2018) [14]	MRI	Three subtypes: -Internal -External -Adenomyoma	Based on Sampson’s observation as well as clinical observations	Different subtypes appear to have different pathogenesis, symptomatology, and severity
Gordts et al. (2018) [5]	MRI/TVUS/hysteroscopy	Important parameters to be included in a classification system: Affected area (inner or outer myometrium), localization (anterior, posterior, or fundus), pattern (diffuse or focal), type (muscular or cystic), volume or size (expressed as <1/3, <2/3, >2/3 or in cm)	These parameters are potentially related to symptomatology and/or severity	Included parameters are important for accurate diagnosis and, through grading, may be associated with disease severity.
Van den Bosch et al. (2019) [37]	TVUS	Location (anterior, posterior, left or right lateral side, or fundus), differentiation (focal, diffuse, or mixed type), cysticity (cystic or non-cystic), uterine layer involvement ( I: involving inner/sub-endometrial myometrium; II: involvement of middle myometrium; III: involvement of outer/sub-serosal myometrium), extent (<1/4, ≥1/4 but ≤1/2, >1/2 myometrium), and size.	Based on consensus among sonographers, and consistent with the previous MUSA consensus.	A welcome first step towards an internationally accepted classification and reporting system
Kobayashi and Matsubara (2020) [20]	MRI	Five main categories: (1) affected area (internal vs. external), (2) pattern (diffuse, focal); (3) size (<1/3, <2/3, or >2/3 of uterine wall); and (4) localization (anterior, posterior, left lateral, right lateral, and fundus); (5) concomitant pathologies (none, PE, OE, DE, UF, others)	Adopted from previous proposals of classification	Combined all important features of adenomyosis that may be useful for proper classification
Exacoustos et al. (2020) [25]	TVUS	Type (focal, diffuse, or adenomyomas), Extension of the lesion in the myometrium	Empirical observations	These variables seem to correlate with the severity of symptoms and infertility

Abbreviations used in the table: DE: deep endometriosis; JZ: junctional zone; MRI: magnetic resonance imaging; OE: ovarian endometrioma; PE: peritoneal endometriosis; UF: uterine fibroids.

## Data Availability

Not applicable.
